# Immunohistochemical Expression Analysis of Caldesmon Isoforms in Colorectal Carcinoma Reveals Interesting Correlations with Tumor Characteristics

**DOI:** 10.3390/ijms24032275

**Published:** 2023-01-23

**Authors:** Alya R. Alnuaimi, Justus Bottner, Vidhya A. Nair, Nival Ali, Razaz Alnakhli, Eva Dreyer, Iman M. Talaat, Hauke Busch, Sven Perner, Jutta Kirfel, Rifat Hamoudi, Wael M. Abdel-Rahman

**Affiliations:** 1 Sharjah Institute for Medical Research, University of Sharjah, Sharjah 27272, United Arab Emirates; 2 College of Medicine, University of Sharjah, Sharjah 27272, United Arab Emirates; 3 Institute of Pathology, University Hospital Schleswig-Holstein, 23560 Luebeck, Germany; 4 Luebeck Institute for Experimental Dermatology, University of Luebeck, 23562 Luebeck, Germany; 5 Division of Surgery and Interventional Science, University College London, London NW3 2PS, UK; 6 Department of Medical Laboratory Sciences, College of Health Sciences, University of Sharjah, Sharjah 27272, United Arab Emirates

**Keywords:** *CALD1*, caldesmon, chemotherapy, colorectal cancer, invasion, l-CaD, metastasis

## Abstract

Colorectal cancer is a notorious disease, with almost half of the patients succumbing to the disease. The prevalence and incidence rates of colorectal cancer are increasing in many parts of the world, highlighting the need to discover new biomarkers for diagnosis and therapy. Caldesmon (CaD), an actin-binding protein that plays a significant role in controlling cell motility, has emerged as a promising biomarker. The *CALD1* gene encodes CaD as multiple transcripts that mainly encode two protein isoforms: High-molecular-weight (h-CaD), expressed in smooth muscle, and low-molecular-weight (l-CaD), expressed in nonsmooth muscle cells. Most studies have suggested an oncogenic role of CaD in colorectal cancer, but the exact subcellular localization of the two CaD isoforms in tumor cells and stroma have not been clarified yet. Here, we analyzed tissue samples from 262 colorectal cancer patients by immunohistochemistry analysis using specific antibodies for l-CaD and h-CaD. The results showed elevated cytoplasmic expression levels of l-Cad in 187/262 (71.4%) cases. l-Cad was expressed at low levels in the normal colon mucosa and was also consistently expressed in the cancer-associated stroma of all cases, suggesting that it could play a role in modulating the tumor microenvironment. l-CaD expression in cancer cells was associated with preinvasive stages of cancer. Survival analysis indicated that patients with high l-CaD expression in tumor cells could respond poorly to selective chemotherapeutic 5FU, but not combination chemotherapy. h-CaD was expressed in colonic and vascular smooth muscle cells as expected and to a lesser extent in the tumor-associated stroma, but it was not expressed in the cancer cells or normal colon mucosal epithelial cells. Collectively, these data clarify how the expression patterns of CaD isoforms in colorectal cancer can have applications in the management of colorectal cancer patients.

## 1. Introduction

Almost half of the patients with colorectal cancer are destined to die of their disease [1], and that is mainly due to metastasis, which is classically thought to be a late step. Moreover, some authors believe that there are subsets of colorectal cancer that metastasize at a very early stage, adding to the problem of managing this notorious disease [2,3]. The metastasis process is augmented by the acquisition of multiple genetic and epigenetic mutations and the activation of a multitude of signaling pathways. These endow metastasizing cells with the ability to shed their adhesion molecules and acquire mesenchymal markers, which enable tissue invasion in a process known as epithelial-to-mesenchymal transition (EMT), and also enable these cells to survive in a new tissue environment [4,5,6]. Additionally, some of these alterations offer resistance to apoptosis; hence cancer cells become resistant to many therapeutic modalities [6,7,8]. These characteristics highlight the need to discover new, more effective biomarkers of early diagnosis and response to therapy in colorectal cancers.

Caldesmon (CaD) is an actin-binding protein that regulates the functions of the actin cytoskeleton in cell motility, contractility, and cell division [9]. The *CALD1* gene encodes CaD in multiple isoforms. High-molecular-weight CaD (h-CaD; 120–150 kDa) is restricted to the smooth muscle cells of visceral and vascular origin, while the low-molecular-weight CaD (l-CaD; 70–80 kDa) isoforms are expressed in nonsmooth muscle cells [10,11,12]. Although h-CaD is used as a specific diagnostic marker for tumors of smooth muscle or those of myofibroblast origin, l-CaD has attracted attention for its role in the development and progression of many types of cancer including colorectal cancer [13].

In transformed cells, CaD is found mainly in the podosome core domain with short F-actin bundles and appears to play an important role in the structure and function of the podosome. CaD was associated with the high motility of the podosomes of the transformed cells. In contrast, the stable adherence of the focal adhesions of normal cells was suggested to be due to the lack of this system [14]. The podosomes of transformed cells are the most dynamic adhesive structures with high motility and short half-life, leading to metastasis and invasion. In contrast, focal adhesions of normal cells were incapable of performing these functions [15]. These findings suggest that CaD exerts contrasting functions in normal and transformed cells. This possibility has attracted the interest of cancer researchers to explore the role of CaD in cancer development. Currently, most of the available data on the role of CaD in carcinogenesis suggests that CaD, particularly l-CaD, plays an oncogenic role in many types of cancer such as breast cancer [16] and colorectal cancer [17].

CaD can exert its oncogenic role via association with transforming growth factor beta (TGF-β), an important mediator of EMT [18,19]. The *CALD1* gene has been associated with TGF-β signaling activation, stromal invasion by malignant cells, and marked angiogenesis in the consensus molecular subtype 4 of colorectal cancer [20]. CaD, particularly l-CaD, can stimulate endothelial cell migration and promote tumor angiogenesis in human tumors [18,21] including stage III/IV mismatch repair-proficient colorectal cancer [22]. An important role of CaD in multiple types of cancer is highlighted by the finding of tumor-specific splicing variants of the *CALD1* gene in cancer tissues including colon, urinary bladder, and prostate cancer [23]. This finding implies that CaD can potentially expand its functions and/or exert altered functions in tumors compared to normal tissue. Moreover, CaD is associated with resistance to multiple forms of cancer therapy including hormonal therapy, immunotherapy, chemotherapy, and radiotherapy [13].

Overall, the above data suggest that CaD is an attractive new candidate to be analyzed for its potential application as a marker of diagnosis and therapy in colorectal cancer. However, some studies, particularly transcriptomic-based analyses, referred to the *CALD1* gene and did not indicate which specific transcript or isoform was involved. There are also contrasting data on the role of CaD in colorectal cancer, as it has been shown to exert tumor suppressor functions in the HCA7 colon cancer cell line [24]. Although HCA7 is known to have unclassical characteristics compared to other colorectal cancer cell lines [25,26], and there was no clinical correlation in that study, these data cast some doubt regarding the role of CaD in colorectal cancer. Furthermore, the expression patterns and subcellular localization of both h-CaD and l-CaD in colorectal cancer tissue have not yet been studied. Therefore, this study set out to address these questions and analyze the expression of both l-CaD and h-CaD in formalin-fixed, paraffin-embedded tissues derived from a large cohort of colorectal cancer cases and to find out if there is any relationship between CaD expression and the clinicopathological characteristics of these tumors.

## 2. Results

### 2.1. l-CaD and h-CaD Expression in Colorectal Cancer and Normal Colon Mucosa

**Figure 1:**
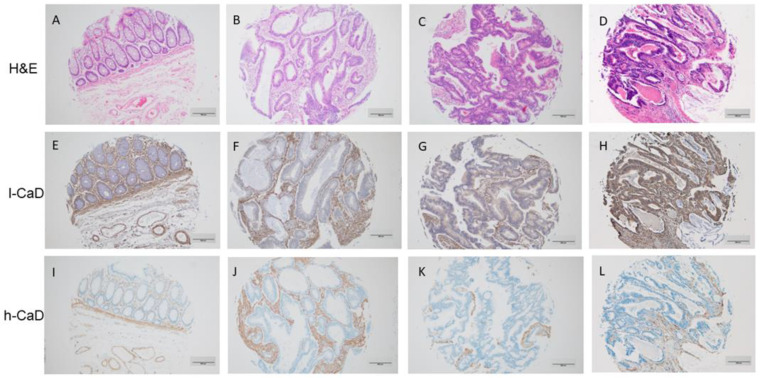
Staining patterns for l-caldesmon (l-CaD; **E**–**H**), and h-caldesmon (h-CaD; **I**–**L**) in colorectal adenocarcinoma tissue assessed using immunohistochemical staining together with matching hematoxylin and eosin (**H**,**E**; **A**–**D**). l-CaD staining ranged from negative/weak (**F**,**G**) to strong (**H**) cytoplasmic staining of tumor cells. The stroma was consistently positive for l-CaD. Chromogen, DAB; original magnification 100; scale bar = 200 μm.

Immunohistochemistry staining of 262, formalin-fixed, paraffin-embedded tumor tissue samples from colorectal cancer cases was performed using antibodies against h-CaD and l-CaD. The results showed elevated l-CaD expression levels in cancer cells in 187/262 (71.4%) cases (designated as positive). The subcellular localization of l-CaD was cytoplasmic. Normal mucosal epithelial cells showed a low level of expression without any apparent predilection to the luminal surface or crypt bases, and it was also cytoplasmic too. However, h-CaD was not expressed in the cancer cells or the normal colon mucosal epithelial cells.

l-CaD was consistently expressed in the cancer-associated stroma of all cases, suggesting a role in modulating the tumor microenvironment. h-CaD was expressed in the smooth muscle cells of the colon and the blood vessels and, to a lesser extent, in the tumor-associated stroma but not in the cancer cells, as stated above (Figure 1 and Table 1).

### 2.2. l-CaD Expression and Clinicopathological Tumor Characteristics

**Figure 2:**
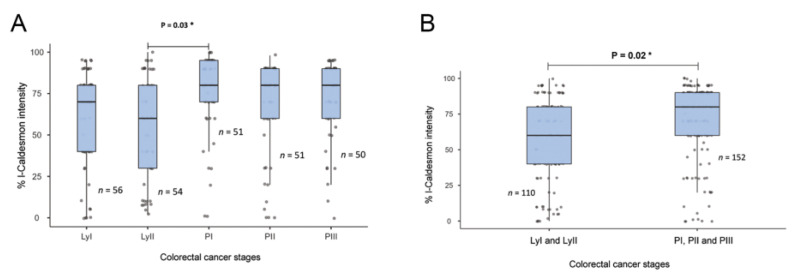
Relationship between l-CaD expression and colorectal cancer tumor stage. (**A**) l-CaD expression by detailed tumor stage; (**B**) l-Cad expression by grouped tumor stage showing a significant relationship between l-CaD and primary tumor stage. Ly, lymph node stage; PI-III, primary tumor stages I-III; * *p* < 0.05.

There was no significant association between l-CaD expression and age, sex, or tumor type. However, l-CaD expression in the colorectal adenocarcinoma cells was associated with the primary stages before lymph node metastasis (Table 1 and Figure 2).

### 2.3. l-CaD and Patients’ Survival

**Figure 3:**
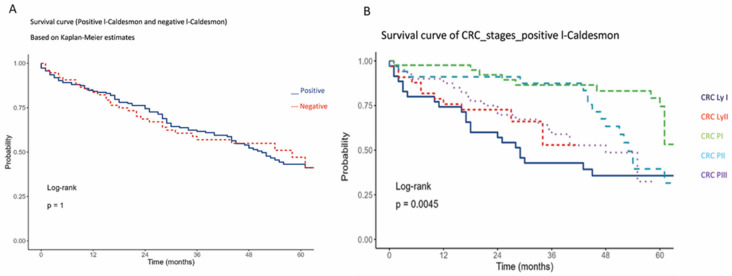
Kaplan–Meier 5-year overall survival analysis of colorectal cancer cases in relation to l-CaD (**A**) and Kaplan–Meier 5-year overall survival analysis of colorectal cancer cases in relation to tumor stage (**B**).

There was no significant association between the 5-year overall survival of our cohort and l-CaD staining status. However, the majority of these patients were under combination chemotherapy, which appeared to affect their survival regardless of the l-CaD status. There was a correlation between tumor stage and survival, with poor 5-year overall survival in the more advanced stages, as expected (Figure 3).

### 2.4. In Silico Analysis of CaD in Colorectal Cancer

**Figure 4:**
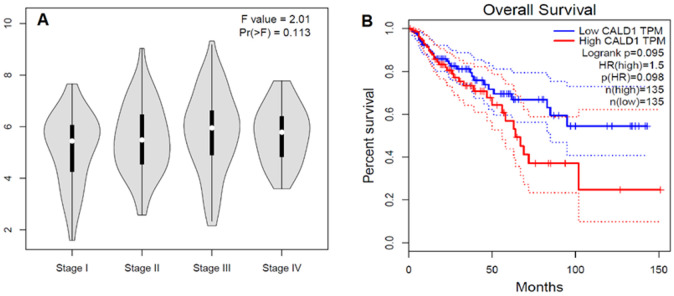
In silico analysis of *CALD1* genes in the TCGA colon adenocarcinoma cases. (**A**) Stage plot showing *CALD1* gene expression in various tumor stages of the colon adenocarcinoma cohort of the TCGA dataset (*n* = 275). (**B**) Kaplan–Meier survival curve shows the overall survival analysis of the cases of TCGA colon adenocarcinoma according to *CALD1* gene expression status. The solid line represents the survival curve and the dotted line represents the 95% confidence interval; HR = 1.5; *p*(HR) = 0.98, *n =* 270. The study was performed using GEPIA at http://gepia.cancer-pku.cn/index.html (accessed on 1 December 2022).

To validate our data in a separate cohort and to find out if these data could be generalized, we searched “The Cancer Genome Atlas” (TCGA) dataset (*n* = 275) for the *CALD1* gene expression in the colon adenocarcinoma cohort. This cohort showed a tendency for *CALD1* gene expression to increase with tumor stage until stage III, and then started to decline (Figure 4A). Similarly, our data also showed more expression in the primary stage before lymph node metastasis. The same cohort showed a tendency for patients with high *CALD1* expression to have poor overall survival (Figure 4B). HR = 1.5; *p*(HR) = 0.98, total cases = 270.

## 3. Discussion

The literature data suggest that CaD plays an important role in cancer development and progression, but the expression pattern and subcellular localization of both h-CaD and l-CaD in colorectal cancer have not been studied in tumor tissue thus far. Here, we analyzed a large cohort of colorectal cancer cases and found that cancer cells expressed l-CaD at various levels but not h-CaD, which is expressed only in the smooth muscle of visceral and vascular origins. Interestingly, l-CaD was overexpressed consistently in the tumor-associated stroma in all cases. l-CaD expression was associated with the pre-metastatic stage, while it continued to be expressed to a lesser extent in the stage of lymph node metastasis. l-CaD was not related to patient survival in this cohort and did not show a significant relationship with treatment outcomes when combination chemotherapy was used. In contrast, l-CaD expression showed a trend for association with poor survival after 5FU therapy alone, suggesting that it predisposes to resistance to 5FU. In silico, analysis of the TCGA colon adenocarcinoma cohort showed that the stage-specific pattern of the *CALD1* gene expression was consistent with our observation and that the *CALD1* gene was associated with poor overall survival of colon adenocarcinoma cases.

We confirmed that the expressed form of CaD in colorectal carcinoma cells is l-CaD, which was overexpressed in the majority of cases, while h-CaD was limited to smooth muscle cells. This finding is consistent with the exon array analysis-based study, which showed that the tumor-specific *CALD1* variant was a short variant predicted to encode for a putative oncogenic protein with potentially altered functions. The most likely variant in that study was expected to be transcript variant 2, which encodes for l-CaD [23]. This finding may also explain the contrasting role of h-CaD and l-CaD in cell contractility and mobility, as it is known that splice variants can exert antagonistic functions in tumors. The best example here is the case of the B-cell lymphoma-extra (BCL-X) long isoform (BCL-X_L_), which exerts an antiapoptotic function. In contrast, the short isoform, BCL-X_S_, is a pro-apoptotic killer protein [28]. The l-CaD splice variant identified in the above study [23] was shown to be associated with metastatic disease and poor overall survival in colorectal cancer [29]. Moreover, abnormal *CALD1* splicing was associated with the upregulation of l-CaD in other tumors such as glioma tumor tissue [30] as well as in body fluids [31,32].

Our data are consistent with a previous report based on a 2-DE-based proteomics study of colorectal cancer tissues enriched for increased cellularity, which showed a significantly higher expression level of l-CaD in primary colon cancer and liver metastasis than in the corresponding normal tissues. In contrast, h-CaD did not show such a difference [17]. Silencing of l-CaD increased p21^Cip1^ (cyclin-dependent kinase inhibitor 1 or CDK-interacting protein 1) and cleaved poly-ADP-ribose polymerases (c-PARP), suggesting that l-CaD blocks apoptosis and promotes cell cycle progression. Silencing of l-CaD also decreased phosphorylated mammalian target of rapamycin (p-m-TOR) and nuclear factor kappa B (NF-κB), two important oncogenic signaling pathways that could exert immune modulation in favor of invasive cancer cells [33,34,35]. Taken together, these data show that l-CaD can interact with multiple signaling pathways that promote tumor progression and resistance to therapy. A bioinformatics-based study showed that *CALD1* was upregulated in early-onset colorectal cancer [36], which is a heterogenous group, most of which share a poor prognosis compared to old-age cancers. However, there were no young-age cancers in our cohort to test this possibility.

We noticed that there was a stage-specific variation in the expression of l-CaD in colorectal cancer, with higher expression in primary tumor stages compared to the stages of lymph node metastasis. In silico analysis of the *CALD1* gene expression showed a similar pattern in the colon adenocarcinoma cohort of the TCGA database. This expression pattern suggests that the role of l-CaD in a cancer cell is more important at the premetastatic phase and might be involved in the preparation and establishment of the premetastatic niche, facilitated by complex reciprocal signaling pathways among tumor cells from primary sites, immune and myeloid cells from bone marrow, and many types of stromal cells at the premetastatic niche [37]. Interestingly, l-CaD was shown to have a major role in immune modulation in colorectal cancer cells [22]. The l-CaD expression pattern observed in our study is consistent with a previous study of locally advanced, non-metastatic colorectal cancers treated with neoadjuvant chemoradiotherapy including 5-FU, which showed that *CALD1* was among the top genes overexpressed in non-responders [38], most likely via the evasion of apoptosis, as previously shown [17]. In this way, l-CaD may not only cause resistance to chemotherapy, but also prepare the premetastatic cells to survive in the new hostile microenvironment. In conclusion, the question remains why the l-CaD protein and *CALD1* gene are more expressed at the premetastatic phase of colorectal cancer. Future studies might clarify this aspect of l-CaD oncogenic function.

The consistent expression of l-CaD in the tumor stroma is in line with previous reports, which showed that l-CaD is expressed in the cancer-associated fibroblasts (CAFs) and other stromal cell populations where it plays a major role in the tumor microenvironment. A comprehensive in silico analysis using bioinformatics of the poor-prognosis subtypes of colorectal cancer in three common classification systems identified the *CALD1* gene as one of the important stromal markers of poor prognosis [39] in the Human Protein Atlas dataset [40]. In that study [39], the *CALD1* mRNA and the CaD protein were upregulated in CAFs and other stromal cell populations in contrast to the epithelial tumor cells [39]. Although this study did not specify which CaD isoform was detected particularly by expression analysis, we and others have shown that l-CaD, but not h-CaD, is unequivocally expressed in carcinoma cells using multiple techniques [17,22,41], in addition to its expression in the tumor-associated stroma. The poor prognosis of colorectal cancer was linked to TGF-β signaling in stromal cells, thus linking *CALD1* to TGF-β signaling in the tumor stroma [39]. *CALD1* was associated with pro-tumorigenic M2 macrophage infiltration in the tumor microenvironment [22], which is known to exert anti-inflammatory, immunosuppressive, and proangiogenic characteristics [42]. Moreover, *CALD1* showed an inverse relationship with plasma cells, CD8 T cells, CD4 memory-activated natural killer (NK) cells, and dendritic cells [22]. Taken together, these data suggest that l-CaD plays an important role in the tumor microenvironment, although the exact mechanism and detailed signaling pathways remain to be clarified.

In our studied cohort, there was no significant relation between l-CaD expression and patient survival, which might reflect the fact that our cohort received the recommended treatment protocol including combination chemotherapy. However, few cases with high l-CaD expression received 5FU alone and showed a trend to have poor survival that did not reach statistical significance. In the TCGA cohort, there was also a trend of colon adenocarcinoma cases with high *CALD1* expression having poor survival compared to those with low *CALD1* expression. Patients with increased expression of l-CaD in their tumors have a poor response to chemoradiotherapy [17,22,29,38], and in vitro silencing of l-CaD by siRNA induced resistance to 5FU treatment in colon cancer cell lines [17] and in non-small-cell lung cancer cells [43].

## 4. Materials and Methods

### 4.1. Patients and Samples

Two hundred and sixty-two patient samples were collected from University Medical Center Schleswig Holstein-Luebeck, Germany. All patients were diagnosed between 2014 and 2018. Formalin-fixed paraffin-embedded tumor tissues were retrieved from the archives of the Institute of Pathology of the University Hospital Schleswig-Holstein, Campus Luebeck. Tissue samples of primary tumors, lymph nodes, and distant metastases were arranged to create tissue microarrays (TMAs) using a semi-automatic tissue arrayer (Beecher Instruments, Sun Prairie, WI, USA). Each TMA carried up to 54 tumor samples, with up to six normal tissue samples collected from non-tumorous areas of tissue samples. Each sample was a triplet of cores with an area of 1 mm^2^. All of the patient’s clinical data such as sex, age, tumor type (type adenocarcinoma, mucinous adenocarcinoma, and others including signet ring cell carcinoma, serrated adenocarcinoma, mixed adeno-neuroendocrine carcinoma, and combined/mixed carcinoid and adenocarcinoma) were included. The study was conducted according to the guidelines of the Declaration of Helsinki and approved by the Ethics Committee of the University of Luebeck (protocol code 20-267).

### 4.2. Immunohistochemistry

We used anti-h-CaD antibody, clone E89 rabbit monoclonal antibody from Cell Marque-Millipore-Sigma (Burlington, MA, USA), and anti-l-CaD polyclonal rabbit antibody from Invitrogen-Thermo Fisher Scientific Corporation (Waltham, MA, USA). Sodium citrate pH 6.0 was used as the antigen retrieval solution for 5 min at 750 W followed by 5 min at 450 W in a microwave oven. Sections were washed with Tris-buffered saline/Tween pH 7.2. The staining area was manually marked with a Dako pen (Agilent Technologies, Santa Clara, CA, USA) before the staining steps. The detection reagents were from the Mouse and Rabbit Specific HRP/DAB (ABC) Detection IHC Kit from Abcam (Cambridge, UK). The kit has a complete set of reagents including peroxidase- and protein-blocking reagents, biotinylated secondary antibody, streptavidin-HRP, and the DAB substrate. Subsequent steps were performed according to the manufacturer’s protocol. Colorectal cancer tissues stained with the same procedure without the primary antibody were negative.

For analysis of the immunohistochemistry staining results, we adapted the immunoreactive score based on previous recommendations [44]. The scoring was generated by multiplying the staining intensity (0–3, where 0, no staining; 1, mild; 2, moderate; 3, strong) by the percentage of immune-stained tumor cells (0–3, where 0, no positive tumor cells; 1, 1–10%; 2, 11–50%; 3, 51–100%) with a total score ranging from 0–9 (category 0 was negative, categories 1–5 were weak, and categories 6–9 were considered as strong positive). Eventually, for statistical analysis, we found that the most informative categorization was to merge the negative and weak categories and compare them to the strong positive category. Two independent investigators performed the microscopic evaluation of the immunohistochemical staining. A semi-quantitative analysis of staining intensity was conducted using Image J software (https://imagej.nih.gov/ij/index.html, accessed on 10 November 2022).

### 4.3. Data Analysis

For the statistical analysis, we used the statistics interface Jamovi (v1.6, The Jamovi Project, Sydney, Australia, https://www.jamovi.org, accessed on 13 December 2022), which was built on top of the R statistical language (R Core Team (2020). R: A Language and Environment for statistical computing (Version 4.0), https://cran.r-project.org, accessed on 13 December 2022). The following packages were used: Survival (Terry M Therneau (2020). A Package for Survival Analysis in R., https://cran.r-project.org/package=survival, accessed on 13 December 2022). The Kaplan–Meier method and logarithmic rank tests were used to calculate the overall survival (OS) by SPSS. We applied the student’s t-test to compare the expression of low l-CaD expression and high l-CaD expression in different types of colorectal cancer and clinicopathological features. For the correlation of l-CaD expression in different colorectal cancer tissue types, Pearson’s Chi-squared test (χ^2^) and/or Fisher’s exact probability test-two-tailed were calculated. A *p*-value below or equal to 0.05 was considered statistically significant. For the descriptive analysis, the following was used: The Jamovi project (2021), Jamovi. (Version 2.2) [Computer Software], retrieved from https://www.jamovi.org (accessed on 13 December 2022), and R Core Team (2021), R: A Language and environment for Statistical Computing (Version 4.0) [Computer software], retrieved from https://cran.r-project.org (R packages retrieved from the MRAN snapshot 1 April 2021).

## 5. Conclusions

In conclusion, our data clarified which CaD isoform is expressed in colon cancer and its subcellular localization. Colorectal cancer cells overexpress l-Cad to various levels but not h-CaD. Moreover, l-CaD was consistently overexpressed in the tumor-associated stroma, suggesting that it plays a role in modulating the tumor microenvironment. l-CaD expression was high in the pre-metastatic stages, suggesting that it prepares the cells for invasion and metastasis, but its role becomes redundant after the cells have metastasized. I-CaD expression in colon cancer is associated with poor survival after 5FU therapy alone, but l-CaD did not show a significant effect on combination chemotherapy in this study. Thus, l-CaD could be a promising potential biomarker for the diagnosis and response to treatment in colorectal cancer.

## Figures and Tables

**Table 1:** L-caldesmon expression in colorectal cancer cases and its relationship to clinical and pathological characteristics.

		Total (*n* = 262) *	l-CaD Positive *n* = 187 (71.4%)	l-CaD Negative *n* = 75 (28.6%)	*p* Value
Sex	Male	128 (48.9%)	93	35	N.S.
	Female	133 (50.8%)	93	40	
Age (cut-off 80 y)	Old ≥ 79	133 (50.8%)	96	37	N.S.
	Young < 79	129 (49.2%)	91	38	
Age (cut-off 50 y)	Old ≥ 50	255 (79.3%)	182	73	N.S.
	Young < 50	7 (2.7%)	5	2	
Histological Type	Adenocarcinoma	230 (87.8%)	165	65	N.S.
	Mucinous adenocarcinoma	22 (8.4%)	15	7	
	Others ^#^	6 (2.2%)	5	1	
UICC	0, I, II, IIA, IIC	76 (29.0%)	57	19	N.S.
	III, IIIA, IIIB, IIIC, IV, IVA, IVB, IVC	154 (58.8%)	108	46	
T-stage	T1, T2, T3	182 (69.7%)	130	52	N.S.
	T4, T4a, T4b	79 (30.2%)	56	23	
N-stage	N0, N1, N1a, N1b, N1c	178 (67.9%)	125	53	N.S.
	N2, N2a, N2b, X	81 (30.9%)	60	21	
M-stage	M0, M1a	218 (83.5%)	155	63	N.S.
	M1b, M1c	43 (16.4%)	31	12	
Stage Grouping	Lymph node stage I, lymph node stage II	110 (41.9%)	70	40	0.02
	Primary stage I, Primary stage II, Primary stage III	152 (58.0%)	117	35	

Table Footnote. * Differences in the total number were due to missing data. ^#^ Other histological types were signet ring cell carcinoma, serrated adenocarcinoma, mixed adeno-neuroendocrine carcinoma, mucinous carcinoma, combined/mixed carcinoid and adenocarcinoma, and in situ adenocarcinoma. Tumor stages at the time of tumor diagnosis were re-assessed according to the 8th edition TNM classification for tumor staging. Abbreviations: l-CaD, low molecular weight caldesmon, N.S., non-significant (>0.05); TNM, T: tumor, N: lymph node; M: metastasis; UICC, Union for International Cancer Control. A detailed explanation of the TNM stages can be found in the 8th edition of the American Joint Committee on Cancer (AJCC) staging manual [27].

## Data Availability

The TCGA data were extracted using the GEPIA website at http://gepia.cancer-pku.cn/detail.php?gene=cald1 (accessed on 1 December 2022).
